# Effectiveness of behavioral-cognitive group therapy on depression, anxiety, and stress of patients with coronary heart disease

**Published:** 2015

**Authors:** M Aghaei, E Samkhaniyan, A Mahdavi, J Faraji, Z Roshandel

**Affiliations:** * Department of Psychology, Faculty of Psychology and Educational Sciences, University of Tehran, Tehran, Iran; ** Health Psychology, South Tehran University; Student Health Psychology, Department of Education and Psychology, Islamic Azad University, Karaj, Iran; *** Payame Noor University, Department of Psychology, Faculty of Psychology and Educational Sciences, University of Payam Noor, Tehran, Iran; **** Clinical Psychology, Islamic Azad University Saveh Branch, Iran; ***** Clinical Psychology, Department of Education and Psychology, Islamic Azad University, Karaj, Iran

**Keywords:** group therapy, cognitive-behavioral, anxiety, stress, coronary heart disease

## Abstract

**Objective.** An appropriate psychological intervention to promote the level of mental health of patients with a coronary heart has a great importance. The existing investigation aimed to study the effectiveness of the behavioral-cognitive group therapy on depression, anxiety, and stress of patients with coronary heart disease.

**Methodology.** The current study was quasi-experimental with a pretest-posttest that used a control group. Hence, 30 of the patients with coronary heart disease in Shahid Rajaee heart center in Tehran chose to use the convenience sampling method and were put in an experimental group and a control group. Both groups were pretested by using a demographic questionnaire, and scale of depression, anxiety, and stress DASS-42. Afterwards, the experimental group was trained for eight sessions of cognitive-behavioral club therapy and the control society gained no intervention. Later, both groups were post-tested, and the acquired information were analyzed by using inferential and descriptive statistical methods accompanied by SPSS 21 software.

**Findings.** The results indicated that the cognitive-behavioral group therapy training significantly reduced depression, anxiety, and stress in patients with coronary heart disease.

**Conclusion.** What should be understood from this study is that the cognitive-behavioral group therapy training had a great positive impact on the decrease of depression, anxiety, and tension in patients with coronary heart disease, since it had an economic cost and a great acceptability by the cases, especially when it was performed in a group.

## Introduction

In the recent decades, cardiovascular disease has been the most common cause of death and the most significant cause of disability in the society. Also, coronary heart disease has been one of the unknown types in the meantime [**1**]. Coronary heart disease led to more than 65000 deaths in 2010. In other words, it was one of the most important causes of death in 2010 [**2**]. This disease results in a reduction of the physical ability, personal and social relationships disorders, ability to perform duties and economic problems [**1**]. 

During the recent years, there have been a variety of studies in the field of etiology and cardiovascular diseases to show the significance of the present issue. The studies of psychological disorders in cardiovascular disease frequently mentioned that this type of sickness is accompanied by depression, stress, and anxiety [**3**]. The risk of cardiovascular events increases when patients with coronary heart disease and even ordinary people have depression [**4**]. Moreover, anxiety and stress were introduced as significant predictors in patients with coronary heart disease [**5**]. Some of the researchers believed that each of the symptoms of depression, anxiety, and stress could be considered as some of the factors affecting the cardiovascular diseases. It was shown that it could decrease duration and prognosis of coronary heart disease and the quality of life of the patients [**6**]. For instance, it was expressed that the creation of depression is deemed as one of the most significant predictors of coronary heart disease [**1**]. It has been approved that depression in patients with coronary heart disease has a substantial impact on the reduction of the quality of life, inappropriate prognosis, and increase of risk of death [**7**]. In other words, the increase in the possibility of mortality in patients with coronary heart disease is in a direct relationship with the extent of depression. Similarly, different studies indicated that the level of anxiety and stress is considered a significant predictor of the rate of coronary heart illness, which reduces the quality of life and increases the risk of death in these patients [**9**]. This happens while patients with chronic diseases such as heart diseases are more vulnerable to the danger of psychological stresses and anxiety due to the lack of adaptive defensive mechanisms and employment of inappropriate methods of coping with stress [**8**]. Therefore, paying a particular attention to the psychological disorders of investigating the strategies or interventions to reduce the conditions has an important significance.

The behavioral-cognitive therapy is among the therapeutic interventions whose effectiveness was approved in a variety of scopes. Behavioral-cognitive therapy is a kind of treatment that emphasizes the way of one’s thinking. In other words, emotions and feelings of a person are influenced by his/ her way of thinking and help the person perceive emotions, feelings and attitudes affect his/ her behaviors [**8**]. Behavioral-cognitive therapy is short-term most of the time, the person learning how to identify and change destructive and annoying thought patterns and emotions that have an adverse impact on his/ her behaviors during treatment, which is usually of eight to twelve sessions [**10**]. According to the behavioral-cognitive therapy, it was emphasized that it is important to discuss the concepts operationally and to validate the treatment experimentally. The major part of the treatment is based on the approach of “Here and Now” and it is assumed that the primary purpose is to help the patient create desirable changes in his/ her life [**11**]. Therefore, during the treatment, people learn to control their thoughts and identify the causes of the feelings and actions. Moreover, opportunities for new adaptive learning and creating changes in the external environment in clinical scores are provided for them [**12**].

It seems that the behavioral-cognitive therapy has a great impact on the reduction of psychological disorders in patients with a coronary disease such as depression, anxiety, and stress; thereby the present research studied the effectiveness of behavioral-cognitive group therapy on depression, anxiety, and stress of the patients with a coronary heart disease.

## Methodology

This study was quasi-experimental with a pretest-posttest that used a control group. The population consisted of all the patients with a coronary heart disease who referred to Shahid Rajaee Health Hospital in Tehran from October to November in 2015. According to the fact that the minimum sample population in the experimental studies should be of 15 individuals, a 15-individuals sample size (n = 15) was chosen for each of the groups. The inclusion criteria of the present study were the diagnosis of a coronary heart disease based on the diagnosis of cardiologists, informed happiness and willingness to partake in the research, ability to take part in the sessions and cooperation in doing tasks, eagerness to cooperate in completing instruments, physical and psychological stability (no apparent physical or mental signs which could intervene during the sessions, such as shortness of breath) and the age ranging between 20 and 45. Also, the patients would be neglected if there was no possibility to continue the study due to the existence of a physical or psychological illness or existence of any cognitive disorder or impaired cognitive function. This way, some of the patients in Shahid Rajaee Heart Hospital were selected and were placed into two groups of control and cognitive-behavioral therapy accidentally.

The implementation method meant that the sampling was conducted of all patients in the hospital who referred to Shahid Rajaee Heart Hospital. Afterwards, 30 individuals were randomly selected and placed in two different groups (15 in each group) after having ensured the inclusion and exclusion criteria. Some explanations were provided about the treatment sessions and the research questionnaire and presented to the patients. In the case of the individual’s approval to participate in the study, he/ she would be randomly placed in one of the groups. Prior to the implementation of the research, in order to observe the ethical principles and to ensure the attendance to meetings, informed consents were obtained in addition to explaining the investigation and its positive impacts, then the experimental group was trained for eight sessions of cognitive-behavioral group therapy, and the control group received no intervention. Finally, both groups were post-tested. The employed protocols for the behavioral-cognitive group therapy sessions are mentioned in **Table 1**.

The instruments used in the present research consisted of a demographic questionnaire, and scale of depression, anxiety and stress DASS-42.

Demographic questionnaire: this questionnaire was formulated to receive the personal information of the participants. Characteristics such as age, gender, education, and marital status were questioned in the survey.

Depression, anxiety, and pressure scale (DASS-42): depression, anxiety, and stress level (DASS-42) were developed in 1995 by Lovibond and Lovibond (Henry & Crawford, 2005). This level consisted of two forms. The short-form consisted of 21 items that evaluated each of the parameters of depression, anxiety, and pressure by seven expressions. The way of responding was based on 4-choice questions (0 = never to 3 = so much). Item distribution was the following: 14 questions for depression, 14 questions for anxiety, and 14 questions for stress. The long-form consisted of 42 items (14 parameters). This scale was based on a Likert scale including no, low, medium, and high elements. The least score for each question was zero, and the maximum score was three. Eventually, the summation of the scores of depression, anxiety, and stress was calculated for each patient. The reliability of the measure was calculated to be 0.91, 0.84, and 0.90 for the variables of depression, anxiety, and stress, respectively by using Cronbach’s alpha method. The short-form of the scale was validated [**13**]. For the Iranian population and the internal consistency of this scale, the values were 0.77, 0.79, and 0.78 for the variables of depression, anxiety, and stress, respectively by using Cronbach’s alpha method. Moreover, Hanif N, Bahraminezhad N, Mirza Khalil Abadi T, Ahmadi F, Khani M, Taran L [**14**] conducted a study to calculate the reliability of the scale as being 0.95, 0.89 and 0.99 for the variables of anxiety, stress, and depression, respectively. 

The Statistical Package for Social Sciences (SPSS-21) software was used to analyze the obtained data. To analyze the research data, based on descriptive statistics, indices of average standard deviation, frequency, and frequency percentage were used. Moreover, based on the inferential statistics, the Analysis of Covariance (ANCOVA) was used.

**Table 1:** Cognitive-behavioral group therapy training protocol

Session	Subject
First	Referral of group members, being familiar with the group policy, introduction of depression, anxiety and stress, and being aware of their physical effects
Second	Recognizing negative thoughts, way of creating these thoughts, learning to overcome negative thoughts
Third	Training to overcome dichotomous thinking, arbitrary interpretations, unbalanced judgments, immediate conclusion, mind-reading, wrong impressions
Fourth	Training to overcome extreme generalization, labeling, inexact term, exaggerated generalization, absolutism, feeling guilty, mental filtering
Fifth	Training to overcome zooming in and out, tragic consequences, not being disastrous, t split swiftness, too much attention to negative situations and personalization
Sixth	Being aware of the time of getting angry, controlling anger and overcoming anger
Seventh	Continuing training, practicing and performing exercises, training relaxation techniques for use in uncomfortable situations
Eighth	Briefly overviewing the sessions and providing feedback to each other, training to transfer data and findings to the external environment of the group

**Research Findings**

The demographic characteristics of the present sample size is listed in **Table 2**.

**Table 2:** Demographic characteristics of the subjects

Variable	Group	Frequency	Frequency percentage	Average and standard deviation
Age	25-30	5	12.5	37.65 ± 6.01
	31-35	12	30	
	36-40	6	15	
	41-45	17	42.5	
Gender	Male	33	82.5	
	Female	7	17.5	
Level of education	Diploma	15	37.5	
	Associate Degree	6	15	
	Bachelor degree	15	37.5	
	Master degree	4	10	
Marital status	Bachelor	5	12.5	
	Married	35	87.5	

**Table 3:** Descriptive statistics of scores of research variables in the two groups according to the pretest and the post test

Component	Index	Experiment		Control	
		Pretest	Posttest	Pretest	Posttest
Depression	Average	17.45	9.05	15.75	15.45
	Standard deviation	2.83	2.39	3.43	3.42
Anxiety	Average	17.30	8.80	15.65	15.65
	Standard deviation	3.24	2.26	2.45	2.49
Stress	Average	20.25	10.20	20.11	19.60
	Standard deviation	2.17	1.60	2.84	2.76

According to **Table 3**, the average scores of the variables of depression, anxiety and stress were decreased in the experimental group compared to the control group.

**Table 4:** Levene test results in order to investigate the default homogeneity of variances of depression, anxiety, and stress in posttest

Variable	Stage	F	Df1	Df2	Sig. level
Depression	Posttest	0.250	1	38	0.620
Anxiety	Posttest	0.418	1	38	0.522
Stress	Posttest	0.569	1	38	0.455

According to **Table 4**, the null hypothesis of the equality of variances of the two groups in variables of depression, anxiety, and stress was approved. In other words, the variances of the two groups were equal to each other for the variables of depression, anxiety, and stress, and there was no significant difference. Therefore, according to the compliance with the Levine defaults, the results referring to the study of the research hypotheses were permissible.

**Table 5:** Results of multivariable ANACOVA on the scores of posttest with a control of pretest in the variables of depression, anxiety, and stress

Test	Value	F	Df	Sig. level	Squared Eta	Power
Pylayy effect	0.896	103.494	3	0.001	0.896	0.95
Wilks Lambda	0.104	103.494	3	0.001	0.896	0.95
Hotelling effect	8.624	103.494	3	0.001	0.896	0.95
Ray’s largest root	8.624	103.494	3	0.001	0.896	0.95

As mentioned in **Table 5**, the significance level of all the tests (P < 0.001) revealed that there were variations among the two groups in at least one of the dependent variables (depression, anxiety, and stress). According to the squared eta, 0.89 percent of the observed differences among the individuals were related to the impact of the independent variable (i.e. intervention method). On the other hand, since the statistical power was equal to 0.95 (greater than 0.80), the sample size was admissible. The results related to the significant difference of each of the dependent variables were mentioned as it follows.

**Table 6:** Results of multivariable ANACOVA in order to investigate the effectiveness of behavioral-cognitive group therapy training on depression, anxiety, and stress in posttest

Index	Sum of squares	Df	Mean Square	F	Sig. level	Squared Eta
Depression	409.601	1	409.601	46.896	0.001	0.552
Anxiety	469.225	1	469.225	82.644	0.001	0.685
Stress	883.601	1	883.601	173.076	0.001	0.821

Based on **Table 6**, since p < 0.001, the hypothesis related to the differences between depression, anxiety, and stress between the two groups was approved. Moreover, it could be expressed as 0.55 percent of the change in the score of depression, 0.68 percent of the change in the score of anxiety and 0.82 percent of the change in the score of stress being due to the independent variable (behavioral-cognitive group therapy training). Therefore, it could express that the behavioral-cognitive group therapy training led to a reduction of depression, anxiety, and stress in patients with coronary heart disease.

## Conclusion

According to the present study on the effectiveness of behavioral-cognitive group therapy training on depression, anxiety, and stress in patients with coronary heart disease, the results indicated that that behavioral-cognitive group therapy training had a significant impact on depression, anxiety, and stress in patients with coronary heart disease. This finding is in line with the studies conducted by Khodaie S, Khazaie K, Kazemi T, Ali Abadi Z, Bayazi MH, Bigdeli I, Rahimian Bougar E [**11**,**13**,**15**].

In explaining the effectiveness of the behavioral-cognitive therapy on the reduction of depression, Khodaie S, Khazaie K, Kazemi T, Ali Abadi Z [**11**] expressed that the existence of a critical component like the behavioral activation together with the cognitive treatments results in a reduction of depression initially and a decrease in the depression mood in case such treatments are continued. Therefore, it can be deduced that paying attention to negative attitudes and emotions leads to a depressed mood. Using active and productive behaviors, dysfunctional cognitive changes and increase in self-efficacy take place. As a result, the depressed mood changes to balanced mood.

Also, the finding of the impact of behavioral-cognitive group therapy on the reduction of anxiety was in line with other studies such as the ones conducted by Khodaie S, Khazaie K, Kazemi T, Ali Abadi Z, Bayazi MH, Bigdeli I, Rahimian Bougar E [**11**,**13**,**15**]. Bigdeli I, Rahimian Bougar E [**15**] found in their research that individuals with heart disease have anxiety states that could decrease by receiving interventions and support in a variety of therapeutic groups. On the other hand, it could be deduced that most of the people with a heart disease have problems in the field of fear of the immediate consequence of illness. Psychological treatments could control this expected anxiety, and this could explain the findings in the present research. In his investigation, Bayazi (2012) mentioned that patients with coronary heart disease would probably try to change their treatment period and life psychological intervention to manage tension, anxiety, and stress to prevent the further recurrence of coronary heart disease. Psychological interventions emphasized making the patients aware of the role of tension, stress and anxiety in such a disease and the methods of controlling stress and anxiety in life, which could be one of the reasons of reducing stress, anxiety, and tension.

Furthermore, the finding that behavioral-cognitive group therapy reduces stress is in line with the studies of Khodaie S, Khazaie K, Kazemi T, Ali Abadi Z, Bayazi MH, Bigdeli I, Rahimian Bougar E [**11**,**13**,**15**]. According to Bigdeli I, Rahimian Bougar E [**15**], the specific issues related to the coronary heart disease such as the role of the family, could be useful in the treatment of such a disease. Moreover, the patients’ tendency to reduce such positions and promotion of behaviors related to health could elevate the effectiveness of the treatments. Also, it could be mentioned that most of the patients with coronary heart disease have a stress which could be managed by the normalization of weight during the treatment. In explaining the findings, the increase of self-management of stress is the objective of most of the socio-psychological interventions in the field of chronic disease and the achievement of such goals could make these interventions last longer.

**Acknowledgement**

The authors would like to thank the respected authorities of Shahid Rajaee Heart Center for their assistance. Moreover, the authors would like to thank all the participants for their cooperation in the present research.
